# Fracture Behavior and Energy Conversion of Concrete–Rock Composites Subjected to Fatigue Disturbance: Experimental and Numerical Approaches

**DOI:** 10.3390/ma19122517

**Published:** 2026-06-11

**Authors:** Lingfei Zhang, Zhongxin Wang, Jian Cao, Kai Zhang, Zhiqiang Zhao, Shuangming Wei, Xiaojun Li, Gan Liu, Jianshuai Hao, Zihan Zhou

**Affiliations:** 1 State Key Laboratory of Digital Intelligent Technology for Unmanned Coal Mining, China Coal Technology and Engineering Group, Beijing 101320, China; zlfcumtb@163.com; 2 Digital and Intelligent Industry Center, CCTEG Shenyang Engineering Company, Shenyang 110013, China; cjcumtb@163.com (Z.W.); au3020@163.com (K.Z.); 15142439578@163.com (Z.Z.); 13610880510@163.com (X.L.); 15524023919@163.com (G.L.); 3 School of Mechanics and Civil Engineering, China University of Mining and Technology, Beijing 100083, China; bqt2300604005t@student.cumtb.edu.cn; 4 School of Safety Science and Engineering, Xinjiang Institute of Engineering, Urumqi 830023, China; weishuangming@163.com; 5 College of Geological Engineering and Geomatics, Chang’an University, Xi’an 710064, China

**Keywords:** rock–concrete composite, interface angle, fatigue disturbance, fracture behavior, mechanical performance, energy conversion

## Abstract

Rock–concrete composites are critical load-bearing elements in geotechnical engineering applications such as slope support. Their mechanical response and damage evolution after fatigue disturbances, such as blasting and mechanical operations, govern the long-term stability and safety of engineered structures. To fully capture these complex behaviors, this study presents a novel multi-scale approach by integrating uniaxial compression tests with three-dimensional digital image correlation and discrete element modeling. This combined experimental–numerical framework is employed to systematically examine the macro- and meso-scale mechanical behavior, crack evolution, and energy response of composites with varying interface angles after quasi-static cyclic loading. The results reveal that as the interface angle increases, the peak strength declines markedly while the brittleness index increases, reflecting a distinct transition in the failure mode from plastic-dissipation-dominated to elastic-energy-storage-dominated. Consequently, the dominant failure mechanism shifts from tensile to shear-slip control. Furthermore, fatigue disturbances exacerbate material degradation, inducing a composite “interface shear–end tension” failure in specimens with higher interface angles and significantly raising the proportion of shear cracks. Energy analysis indicates that cyclic loading enhances the elastic energy storage capacity, and the energy conversion threshold rises continuously with the interface angle. These findings clarify the multi-scale control mechanisms of interface geometry on fatigue-induced failure, providing a theoretical foundation for predicting fatigue life and enabling early pre-warning of failures in rock–concrete engineering structures.

## 1. Introduction

The rock–concrete composite is a critical load-bearing element in rock engineering applications such as open-pit slope support. During its service life, this structure is frequently subjected to cyclic dynamic loads originating from diverse sources, including blast-induced vibrations, mechanical operations, vehicular traffic, and rainfall-induced seepage in open-pit mine slopes [1,2,3,4]. Such cyclic loading not only redistributes stresses within the structure but also promotes progressive accumulation of fatigue damage, which may degrade the rock–concrete interface, initiate and propagate cracks, and drive complex energetic responses. These processes substantially undermine the long-term stability and safety of the structure [5,6,7]. Therefore, systematic investigation of the mechanical behavior and energy response of rock–concrete composites under fatigue disturbances is essential for elucidating slope–support damage mechanisms and for improving fatigue resistance and operational safety.

Currently, extensive research has explored the interfacial mechanical behavior of rock–concrete composites under static loading [8,9,10,11]. Previous studies have systematically examined the effects of loading rate [12], interface roughness [3,13], and inclination angle [14,15] on the macroscopic tensile and shear performance of the interface. However, these static-focused studies cannot fully represent the actual service conditions of open-pit engineering. In practical engineering, rock–concrete composites are frequently subjected to cyclic loads (e.g., blasting, mechanical operations, and mining tremors) that cause cumulative fatigue damage [16,17,18,19]. While preliminary studies have investigated uniaxial fatigue [20] and pre-fatigue fracture behavior [21,22], the multi-scale damage mechanisms under fatigue disturbances remain poorly understood. Specifically, the coupled effects of varying interface angles and cyclic loading on full-field crack propagation and meso-scale energy evolution represent a critical research gap. The novelty of this study lies in addressing this gap by bridging macroscopic strain localization with mesoscopic force-chain and energy evolution.

Conventional microscopic observation techniques, while widely used to characterize fatigue performance, are limited in directly resolving interfacial crack initiation and propagation because microcrack tips frequently fall below their spatial resolution [23,24,25]. Back-face strain measurements can indirectly indicate crack development but cannot unambiguously capture interfacial damage and may lead to misinterpretation [26]. To overcome these limitations, non-contact full-field measurement methods such as digital image correlation (DIC, a non-contact optical measurement technique used for full-field surface strain and displacement analysis) have been increasingly adopted to identify crack initiation and propagation paths with high precision [27,28]. Furthermore, numerical tools such as the discrete element method (DEM) and particle flow code (PFC 7.0.13, a software based on the DEM) can simulate material damage accumulation, crack evolution, and energy redistribution processes from a mesoscopic perspective, providing an effective means to reveal the microscopic mechanisms behind macroscopic mechanical behavior [1,29]. Recently, significant advancements have been made in understanding the mechanical degradation and damage evolution of rock and concrete materials under complex loading conditions. For instance, extensive studies have investigated the cyclic fatigue behavior, crack propagation characteristics, and energy dissipation mechanisms of related rock–concrete composite structures [30,31]. Furthermore, modern monitoring techniques and advanced experimental setups have been increasingly employed to evaluate the structural integrity and progressive failure patterns of heterogeneous geomaterials under varying stress paths [32,33,34].

To distinctively advance beyond existing static and macroscopic evaluations, this study integrates uniaxial compression testing, 3D-DIC full-field strain monitoring, and PFC2D modeling to systematically examine composites with varying interface angles under quasi-static cyclic loading. Unlike previous works that rely on traditional indirect observations, this combined experimental–numerical approach enables the direct visualization of interfacial crack initiation and the quantitative tracking of energy redistribution. The work aims to elucidate the associated damage mechanisms across macro- and meso-scales, thereby providing a reliable theoretical and experimental basis for optimizing the design, assessing the stability, and enabling early warning of disasters in open-pit slope protection structures.

## 2. Experimental Scheme and Numerical Simulation

### 2.1. Specimen Preparation

The rock–concrete composite specimens consisted of natural rock elements bonded with cast concrete. The concrete was prepared using a mixture of P·O 42.5 ordinary Portland cement, coarse aggregate (well-graded crushed stone with a maximum particle size of 10 mm), and medium river sand (particle size < 4.75 mm). The rock material was blue sandstone sourced from a quarry in Zigong, Sichuan Province, China. Its measured physico-mechanical properties were uniaxial compressive strength = 75 MPa, elastic modulus = 15 GPa, Poisson’s ratio = 0.20, water absorption = 3.35%, and permeability = 0.284 mD.

**Figure 1:**
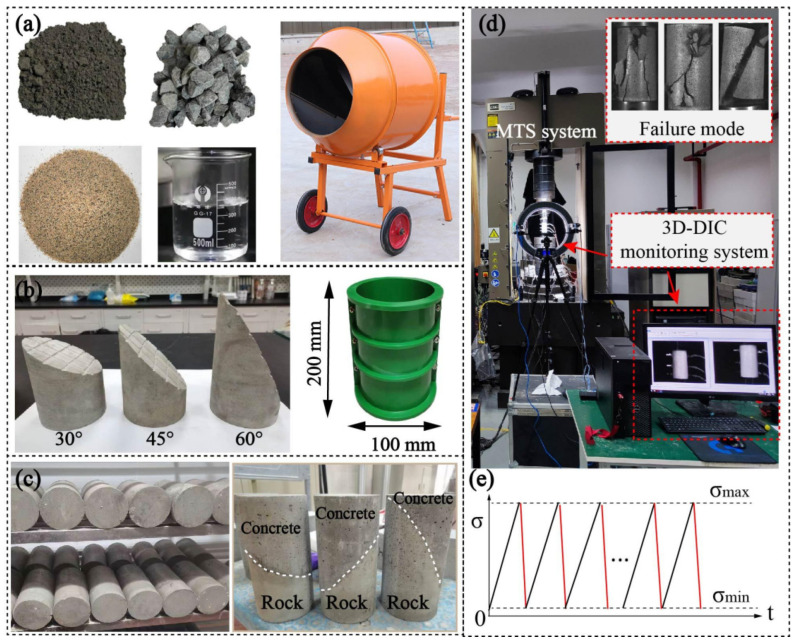
Specimen preparation and experimental process. (**a**) Raw materials of the concrete and specimen preparation. (**b**) Rock specimen. (**c**) Rock-concrete composite specimen. (**d**) MTS servo-hydraulic testing system. (**e**) Stress-controlled cyclic protocol.

The specimen preparation procedure is illustrated in Figure 1a–c. Cylindrical rock cores (100 mm diameter × 20 mm height) were first cut along planes inclined at 30°, 45°, and 60° to the horizontal. These specific angles were strategically selected because they represent the most common inclination ranges of structural planes and potential sliding surfaces in actual open-pit rock slopes. The prepared rock halves were then placed in a plastic mold (inner dimensions: 100 mm diameter × 200 mm height). To ensure strict experimental reproducibility and to control the interfacial mechanical interlocking, standardized rectangular grooves were machined into the rock interface using a high-precision CNC milling machine (Shandong The Dongs CNC Equipment Co., Ltd., Zaozhuang, China). The geometric parameters of the grooves were rigorously quantified, achieving a controlled depth of 2.0 mm, a width of 2.0 mm, and a uniform center-to-center spacing of 20.0 mm. A concrete mix with a mass ratio of cement:coarse aggregate:sand:water = 1425:2367:2892:590 was proportioned, mixed, and poured into the mold. Compaction was achieved by vibrating the filled mold for 120 s. After an initial 24 h air curing period, the specimens were demolded and cured for 28 days. Finally, both ends of each specimen were ground to ensure non–parallelism within 0.02 mm. A total of 18 specimens were manufactured: six for each interface angle (30°, 45°, 60°). For every inclination, three specimens were tested under monotonic static loading and three under quasi-cyclic static loading. Material proportions, fabrication tolerances, and curing conditions were strictly controlled to ensure uniformity and repeatability across all tests.

### 2.2. Testing Apparatus and Experimental Procedure

Quasi-static cyclic loading tests were performed on an MTS SILENTFLO 515 servo-hydraulic testing system (TÜV Rheinland, Cologne, Germany, Figure 1d), which has a maximum axial load capacity of 2000 kN and can apply a dynamic pressure up to 70 MPa. Full-field deformation was monitored using a 3D-DIC system equipped with two high-speed CCD cameras (SVSI, Auburn, AL, USA). The cameras, each with a maximum resolution of 1280 × 1024 pixels and a maximum acquisition rate of 232 fps, recorded the failure process and provided 3D full-field strain maps over the specimen surface. A stress-controlled cyclic protocol was adopted, defined by preset upper and lower stress limits (Figure 1e). The upper limit (σ_max_) was set to 50% of the estimated peak strength (applying 50% σ_max_ ensures sufficient accumulation of fatigue damage while strictly preventing premature failure during the cyclic phase), whereas the lower limit (σ_min_) was held constant at 100 N. Forty loading cycles were applied to induce pre-fatigue damage, rather than testing to failure. Preliminary tests confirmed that energy dissipation stabilizes by 3–40 cycles, achieving the desired steady-state damage phase. For reference, monotonic compression tests were carried out under displacement control at 0.01 mm/min. In the cyclic tests, however, both loading and unloading were force-controlled, using constant rates of 3 kN/s (loading) and 5 kN/s (unloading). All specimen identifiers and corresponding loading parameters are listed in Table 1.

### 2.3. Development and Calibration of the Discrete Element Model

**Figure 2:**
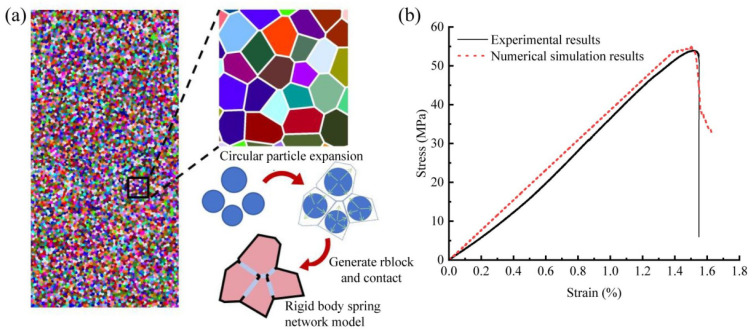
Numerical model and calibration results. (**a**) PFC^2D^ numerical model. (**b**) Comparison of stress–strain curves between numerical simulation and physical experiment.

To investigate the damage evolution and failure mechanisms of rock–concrete composites under fatigue disturbances across different interface angles, a numerical model was developed using the PFC^2D^ in two dimensions based on discrete element theory. The model geometry replicates that of the physical specimens (Φ100 mm × 200 mm) and consists of two distinct cluster-based particle assemblies representing concrete and rock. A smooth-joint contact model was assigned along the predefined dip angles (30°, 45°, 60°) to realistically capture interface behaviors such as slip and opening, as well as the macroscopic frictional and cohesive response (Figure 2a). The meso-scale mechanical parameters used in the model—including particle stiffness, parallel bond strengths, and smooth–joint properties—are summarized in Table 2. Regarding parameter sensitivity, the particle effective modulus and stiffness ratio primarily control the macroscopic elastic modulus and Poisson’s ratio; the parallel bond strengths determine the peak compressive strength of the composite; and the friction angle dictates the post-peak softening behavior. As is well known, DEM parameter calibration often faces the limitation of non-unique solutions, meaning multiple microscopic parameter combinations might produce similar macroscopic stress–strain responses. To effectively overcome this limitation and ensure the physical reliability of the selected parameter set, a multi-objective constraint strategy was adopted for the model’s calibration: we required not only the numerical specimens to highly align with the experimental results in terms of macroscopic responses such as uniaxial compressive strength, elastic modulus, and stress–strain curve morphology (Figure 2b), but also strictly demanded the simulated failure modes and crack propagation paths to be highly consistent with the macroscopic observations from the physical experiments.

## 3. Results and Analysis

### 3.1. Mechanical Properties of the Rock–Concrete Composite

#### 3.1.1. Characteristics of Stress–Strain Curves

**Figure 3:**
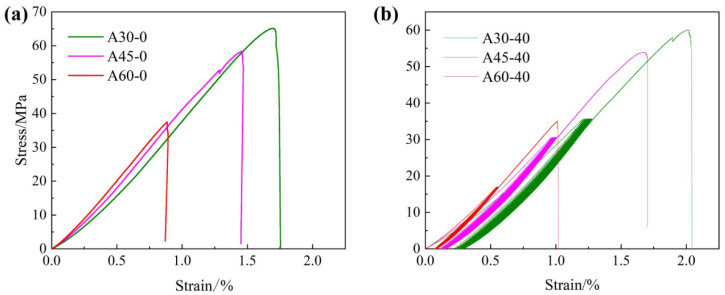
Stress–strain curve. (**a**) Static load test. (**b**) The 40-cycle cyclic loading and unloading test.

Figure 3 presents the characteristic stress–strain responses of the rock–concrete composite specimens under distinct loading modes. Under static loading (Figure 3a), the stress–strain curves for the three interface angles nearly overlap during the initial compaction stage. As loading proceeds into the elastic deformation stage, the curves progressively diverge, demonstrating that interface angles significantly influence the elastic behavior of the composite. Notably, a lower interface angle corresponds to a moderately reduced slope in the elastic region, indicating lower material stiffness and enhanced deformability. Following quasi-static cyclic loading (Figure 3b), the unloading path consistently lags behind the loading path, forming distinct hysteresis loops, and the hysteresis behavior observed in the macroscopic stress–strain curves reflects the continuous energy evolution within the composite. To avoid redundancy here, a detailed quantitative characterization of this behavior, specifically focusing on the evolution of energy dissipation per cycle (E_d_), is further discussed in Section 4.1. Upon commencement of a subsequent cycle, the new loading curve intersects the previous unloading curve, resulting in closed hysteresis loops. Specimens with smaller interface angles develop longer hysteresis loops and accumulate greater plastic strain. Moreover, with increasing cycle numbers, the overall stress–strain curve shifts progressively rightward along the strain axis.

#### 3.1.2. Analysis of Mechanical Parameters

**Figure 4:**
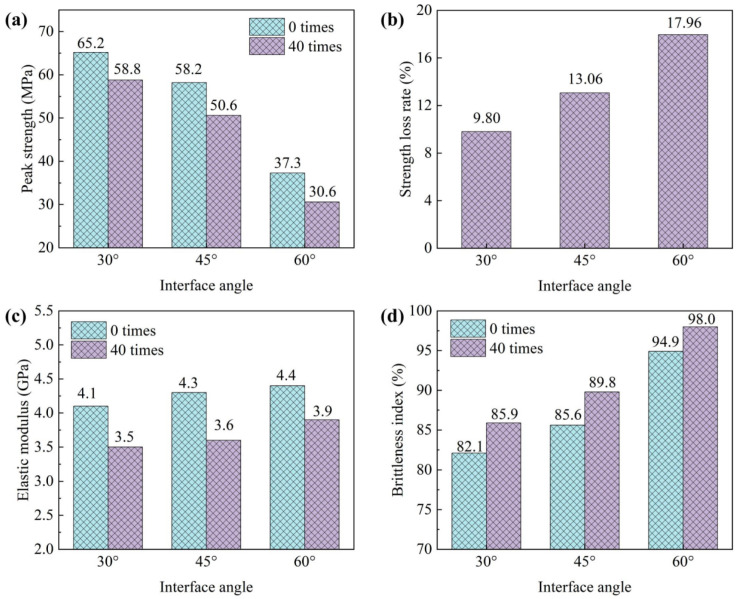
Peak strength and elastic modulus. (**a**) Peak strength. (**b**) Strength loss rate. (**c**) Elastic modulus. (**d**) Brittleness index.

The degradation in mechanical performance of composites with different interface angles under fatigue disturbances is shown in Figure 4a,b. The 30° composite had an initial strength of 65.2 MPa, which decreased to 58.8 MPa after 40 cycles, corresponding to a strength reduction of 9.80%. For the 45° composite, the strength dropped from 58.2 MPa to 50.6 MPa (13.06% reduction). The most pronounced degradation occurred in the 60° composite, with strength declining markedly from 37.3 MPa to 30.6 MPa, representing a loss of 17.96%. These results confirm that the interface angle critically influences mechanical degradation under fatigue disturbances, with greater angles leading to substantially higher strength loss.

As shown in Figure 4c, fatigue disturbances also considerably reduced the elastic modulus. The modulus of the 30° composite decreased from 4.1 GPa to 3.5 GPa (14.6% reduction), that of the 45° composite fell from 4.3 GPa to 3.6 GPa (16.3%), and the 60° composite declined from 4.4 GPa to 3.9 GPa (11.4%). This attenuation is primarily attributed to progressive crack development and interfacial weakening during fatigue disturbances. To evaluate the brittle behavior and assess the potential risk of brittle failure during long-term service, the brittleness index was analyzed. This index was calculated by comparing the actual cumulative elastic strain energy from the pre-peak stress–strain curve with that of an ideal elastic deformation (Appendix A.1). The brittleness indices for composites with different interface angles are presented in Figure 4d. Before fatigue disturbances, the brittleness index increased with interface angle: 82.1% for 30°, 85.6% for 45°, and 94.9% for 60°. After 40 loading–unloading cycles, these values rose to 85.9%, 89.8%, and 90.0%, respectively. This increase indicates that fatigue effects promote crack propagation and strain localization, thereby reducing material ductility and enhancing brittle characteristics.

#### 3.1.3. Evolution of Deformation Modulus Under Constant-Amplitude Fatigue Disturbances

**Figure 5:**
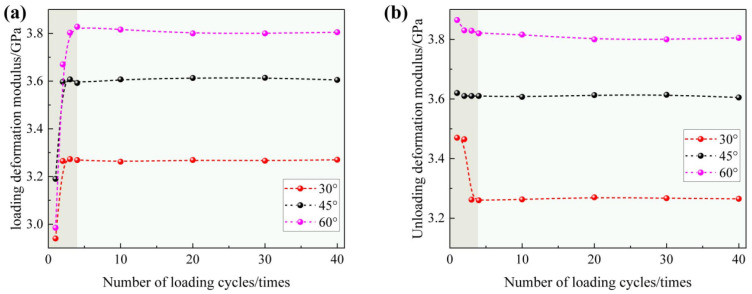
Loading and unloading deformation modulus. (**a**) Loading deformation modulus. (**b**) Unloading deformation modulus.

As detailed in Appendix A.2, the unloading deformation modulus within a single cycle consistently exceeds the loading modulus. Figure 5 shows that the loading modulus increases rapidly during initial cycles and then stabilizes, whereas the unloading modulus first decreases with cycle number before plateauing. In the first cycle, the stress–strain curves form an open hysteresis loop, with the unloading modulus higher than the loading modulus. For example, the initial loading moduli for specimens A30, A45, and A60 were 2.94 GPa, 3.19 GPa, and 2.99 GPa, respectively, while the corresponding unloading moduli measured 3.47 GPa, 3.62 GPa, and 3.87 GPa. This difference occurs because axial compression during initial loading progressively closes inherent micro-fissures. The resulting interlocking of fracture surfaces prevents some fissures from fully reopening upon unloading, leading to accumulated plastic deformation. With continued cycling, the specimens transition into a stage dominated by elastic deformation: the loading and unloading paths converge, forming nearly closed hysteresis loops, and both deformation moduli stabilize around similar values. The stabilized moduli were approximately 3.27 GPa (30°), 3.62 GPa (45°), and 3.81 GPa (60°), showing a clear positive correlation with interface angle (E_30_ < E_45_ < E_60_).

### 3.2. Failure Process and Crack Evolution of the Composite Based on DIC Technique

**Figure 6:**
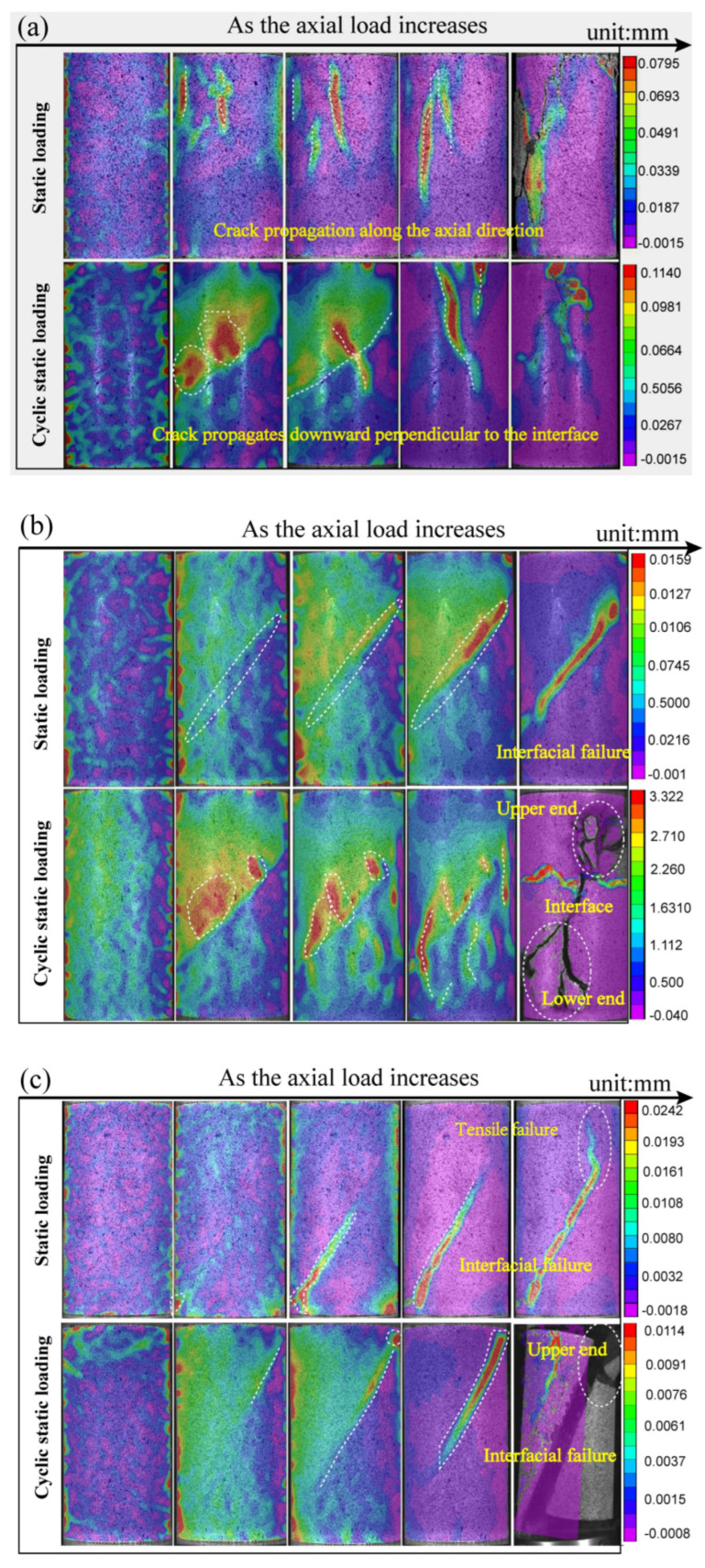
Evolution process of the principal stress and displacement cloud maps of the composite structure during compression: (**a**) 30°, (**b**) 45°, (**c**) 60°.

Figure 6 presents the surface strain contours of specimens under uniaxial compression, with the upper portion representing concrete. For the A30 specimen under static loading (Figure 6a), strain concentration initially emerged on the concrete side, signaling the initiation of vertical cracks. As axial load increased, these cracks propagated downward into the rock, eventually coalescing into a through-going tensile crack that led to failure. By contrast, in the specimen pre-subjected to fatigue, the strain concentration zone was closer to the interface, and the resulting crack propagated rapidly in a direction nearly perpendicular to the interface, forming an oblique crack. This behavior is attributed to pronounced strain concentration at the interface, driven by fatigue disturbances, which in turn guides crack propagation along the path of maximum strain. In later loading stages, additional tensile cracks developed at the specimen’s upper end, ultimately forming an interconnected crack network that caused final failure.

For the A45 specimen (Figure 6b), strain concentration zones under both static and cyclic loading were located near the interface and tended to propagate along it. Under static loading, strain remained largely confined to the interface, resulting in a shear-slip failure mode. In contrast, after cyclic disturbance, strain localized initially on the concrete side, accompanied by a distinct increase in the high-strain (red) zone; cracks subsequently propagated from both interface ends into the rock. Concurrently, strain concentrations of varying intensity developed on the rock side. Once the accumulated internal energy reached a critical level, the rock fractured abruptly, leading to a composite failure mode dominated by interfacial shear-slip coupled with a “shear + tensile” mechanism at the interface ends.

### 3.3. Analysis of Meso-Damage and Crack Evolution in the Composite Based on PFC^2D^

#### 3.3.1. Analysis of Mechanical Response and Meso-Damage Evolution Characteristics

**Figure 7:**
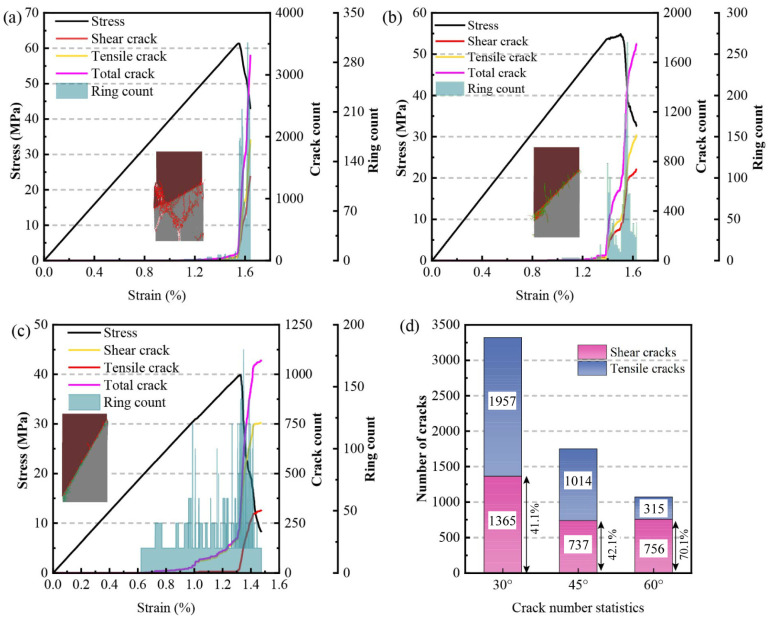
Stress–strain curves, crack numbers, the difference between tensile and shear cracks, crack distribution, and AE counts of composite specimens. (**a**) 30°, (**b**) 45°, (**c**) 60° (**d**) Crack number statistics.

In this section, the numerical AE characteristics are analyzed to evaluate the fatigue damage process. It should be clarified that the reported AE events and counts represent numerical proxies derived from kinetic energy release and micro-cracking within the DEM model, rather than physical acoustic signals measured by sensors in a laboratory setting. Figure 7 illustrates the relationship between crack evolution, numerical AE responses, and the stress–strain behavior of the rock–concrete composites under cyclic loading, based on PFC2D numerical simulations. It should be explicitly noted that the simulated AE activity reported in this study is not derived from experimental measurements using physical sensors; rather, it defined as energy release events triggered by the breakage of parallel bonds within the discrete element model. While this method is widely used in DEM studies of rock mechanics to characterize the temporal sequence of microcrack activity, it does not simulate the amplitude, frequency, or waveform of real physical AE signals. Therefore, it serves solely as a qualitative indicator of mesoscopic damage intensity.

The specimen with a 30° dip angle failed in a tensile-dominated mode (Figure 7a). During the linear elastic stage (strain < 1.2%), tensile and shear cracks appeared sporadically while the simulated AE event count remained below 50. Upon entering the nonlinear stage (strain ≥ 1.2%), tensile-crack propagation accelerated, accompanied by a rise in the AE event count to about 200. At peak strain (~1.6%), tensile cracks coalesced into a penetrating fracture, and the simulated AE count surged above 2000, reflecting the abrupt nature of tensile failure. The specimen with a 45° dip angle exhibited a shear–tensile composite failure (Figure 7b). Below 1.2% strain, interfacial shear cracks initiated first, raising the simulated AE count to approximately 80. Above this strain, both shear and tensile cracks developed continuously, and the ring-down count increased stepwise to around 350. Approaching peak strain (~1.6%), both crack types multiplied, and the AE count peaked near 1600, indicating coupled interfacial slip and tensile rupture. For the 60° specimen, shear-dominated failure was observed (Figure 7c). Shear cracks formed rapidly below 0.6% strain, with simulated AE activity rising above 100 counts at an early stage. Within 0.6–1.2% strain, the AE events remained high (250–300). At peak strain (~1.4%), stress dropped abruptly together with a sharp AE peak exceeding 1200 counts, signaling interfacial shear instability.

A quantitative summary is given in Figure 7d. As the dip angle increased from 30° to 60°, the proportion of shear cracks rose from 41.1% to 70.1%, confirming a transition from tensile- to shear-dominated failure. The simulated AE active phase started earlier and lasted longer at higher angles, suggesting that shear damage develops more progressively. Tensile cracks at the interface ends were universal, affirming the prevalence of stress concentration there. The strong synchrony between the peak simulated AE count and peak stress demonstrates that tracking these numerical AE proxies can serve as a reliable early-warning indicator for imminent damage.

#### 3.3.2. Crack Propagation Laws and Displacement Evolution Process

**Figure 8:**
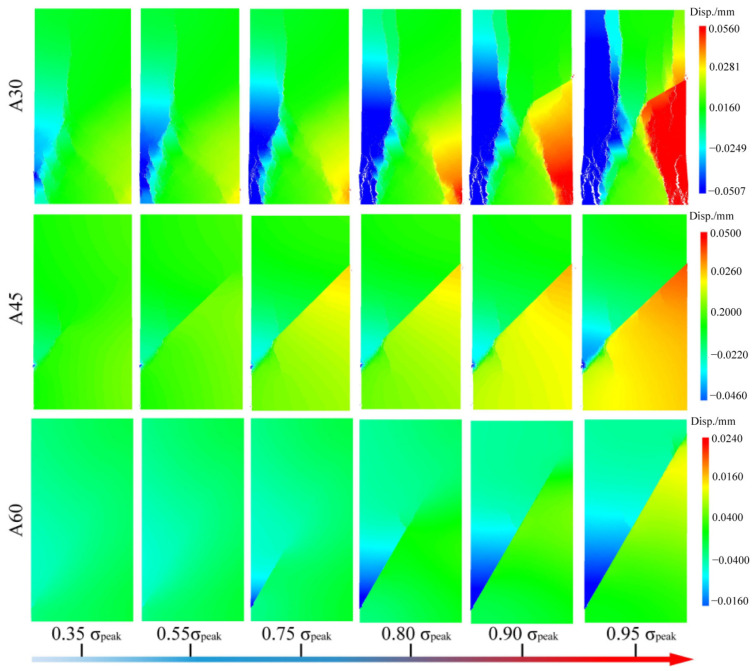
Specimen deformation and evolution across stress stages.

The deformation response and failure mechanisms of specimens subjected to cyclic loading–unloading varied significantly with increasing axial stress. A systematic analysis of displacement evolution in the A30, A45, and A60 specimens under progressive loading (Figure 8) yielded the following observations: at the low-stress stage (0.35 σ_peak_), all specimens exhibited relatively small maximum displacements (A30: 0.0249 mm, A45: 0.0220 mm, A60: 0.0240 mm), with deformation being largely homogeneous and elastic in nature. Upon increasing the stress to 0.80 σ_peak_, the A30 specimen displayed a maximum displacement of 0.0507 mm—a 103.6% increase from the low-stress stage—accompanied by reduced displacement uniformity and emerging localization, signifying entry into the macroscopic nonlinear yield stage. The A45 specimen reached 0.0460 mm (a 109.1% increase), with displacement concentrating notably in the central region, indicative of a shear-dominated deformation mechanism. The A60 specimen showed a distinct spatial pattern, with displacement localized in the upper and lower zones, peaking at 0.0400 mm (a 66.7% increase). As stress neared the peak, the A60 specimen further exhibited nonlinear displacement fluctuations (0.90 σ_peak_: 0.0404 mm; 0.95 σ_peak_: 0.0161 mm) characteristic of the complex mechanical behavior approaching failure. Overall, the deformation mechanism evolved systematically with the interface angle: A30 specimens deformed uniformly and progressively, A45 specimens developed clear shear-localized patterns, and A60 specimens showed pronounced displacement localization. The percentage increases in maximum displacement from 0.35 σ_peak_ to 0.80 σ_peak_—103.6% (A30), 109.1% (A45), and 66.7% (A60)—further quantify the distinct stiffness degradation and damage evolution pathways associated with different interface angles.

#### 3.3.3. Evolution of Contact Force Chain Network

**Figure 9:**
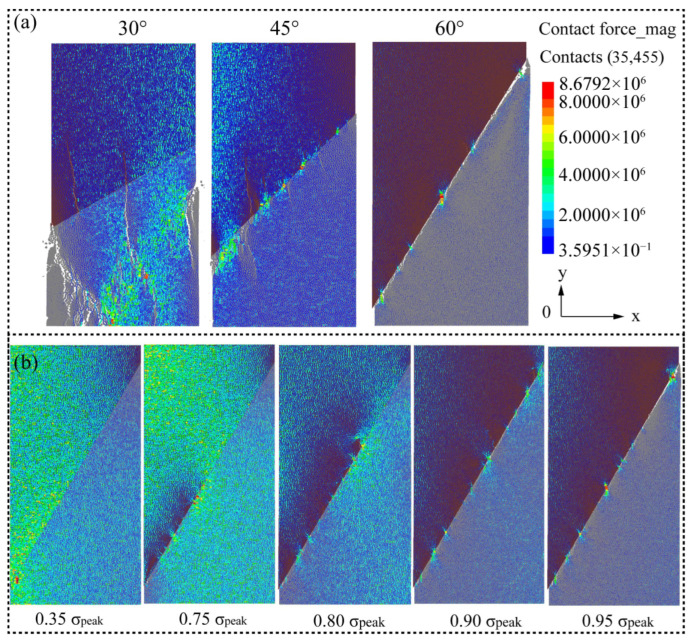
Numerical simulation of force-chain networks: (**a**) effect of interface angle and (**b**) evolution across different stress levels.

Force chains constitute the fundamental meso-scale structures governing load transfer in granular media. Their spatial configuration and dynamic evolution directly determine the intrinsic mechanisms governing interfacial mechanical behavior. To quantitatively analyze force-chain distributions under different conditions, high-capacity force chains (shown in red) are defined as those with contact forces exceeding |F_c_| > 8 × 10^6^ N; the remaining contacts are classified as weak force chains. Figure 9a illustrates the progressive transformation of the force-chain network with increasing interface angle. Initially diffuse and quasi-isotropic at 30°, the network evolves into a highly anisotropic configuration at 60°, where force chains become predominantly aligned normal to the interface. This systematic transition reveals three distinct organizational regimes: force chains maintain multidirectional load transfer with minimal orientational preference (A30). Spatial clustering emerges alongside noticeable directional concentration near interfacial asperities (A45). The network reorganizes into a highly efficient load-bearing skeleton through near-perfect normal alignment (A60). This evolution is governed by interfacial geometry via its control of mechanical interlocking: low angles facilitate distributed load transfer through multidirectional chains, whereas steeper angles progressively constrain chain orientation, ultimately forming specialized, maximally anisotropic load-bearing structures.

Complementing this spatial analysis, Figure 9b depicts the temporal evolution of force chains across increasing stress levels. The mechanical response progresses through four characteristic stages: (1) at 0.35 σ_peak_, a homogeneous network with uniform force distribution facilitates elastic load transfer; (2) at 0.75 σ_peak_, force-chain intensification and a skewed force distribution mark the transition to elastoplastic behavior; (3) in the 0.80–0.90 σ_peak_ range, network reconstruction accompanied by peaked, heavy-tailed distributions coincides with full interlocking development and micro-damage initiation; (4) at 0.95 σ_peak_, extreme force localization into bimodally distributed high-capacity chains creates pronounced spatial heterogeneity that directly precedes macroscopic failure. Together, the spatial and temporal perspectives demonstrate that interface morphology governs macroscopic mechanical properties through its control of force-chain architecture. The established correlation between geometric parameters and force-chain organization provides a mechanistic basis for predicting damage evolution and failure modes in rock–concrete composites.

### 3.4. Discussion of Damage Mechanisms of Composite Post-Cyclic Loading

**Figure 10:**
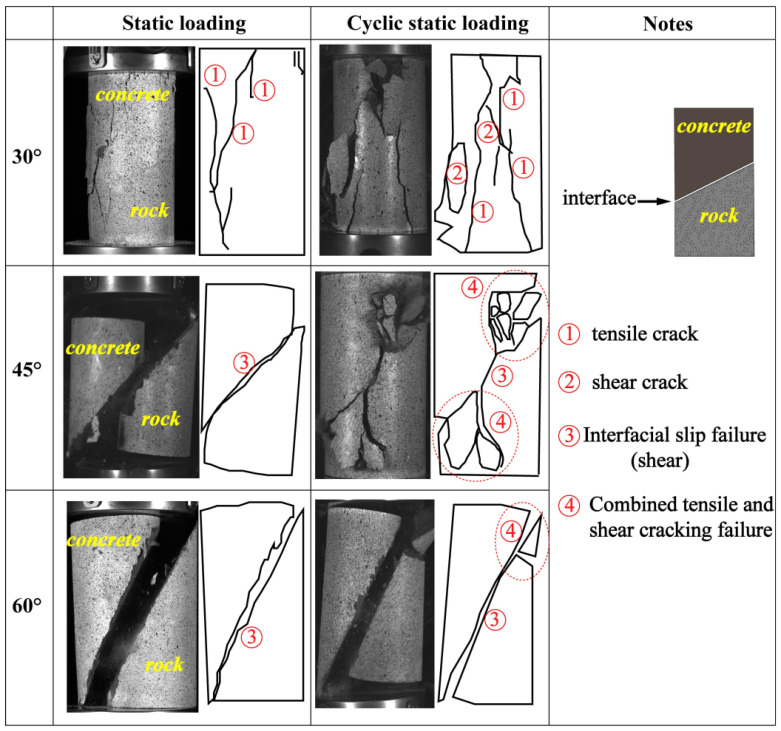
Failure modes.

Analysis of the macroscopic failure modes after cyclic disturbance (Figure 10) identified four characteristic crack-propagation patterns, highlighting the coupled influence of interface angle and cyclic disturbance on failure behavior. In A30 specimens with a 30° interface, static loading produced primarily tensile failure (Type ① cracks), manifesting as one or a few through-going tensile cracks. After quasi-static cyclic loading, tensile failure remained dominant but was accompanied by a significantly denser crack network, resulting in a composite Type ① + ② crack pattern. As the interface angle increased to 45° and 60°, the failure mechanism progressively transitioned from tension-dominated to interface-slip-dominated. Under static loading, A45 specimens failed by shear slip (Type ③ cracks) along the 45° interface, effectively splitting the specimen into two relatively intact portions. After cyclic disturbance, the failure evolved into a composite mode (Type ③ + ④ cracks) that combined interfacial slip with tensile cracking at the specimen ends, producing a distinct wedge-shaped failure zone. Similarly, static loading of A60 specimens induced shear failure (Type ③ cracks) along the 60° interface. After cyclic disturbance, in addition to interfacial slip, a tensile–shear composite failure (Type ③ + ④ cracks) developed in the rock near the upper end, underscoring the aggravating role of fatigue in material damage.

**Figure 11:**
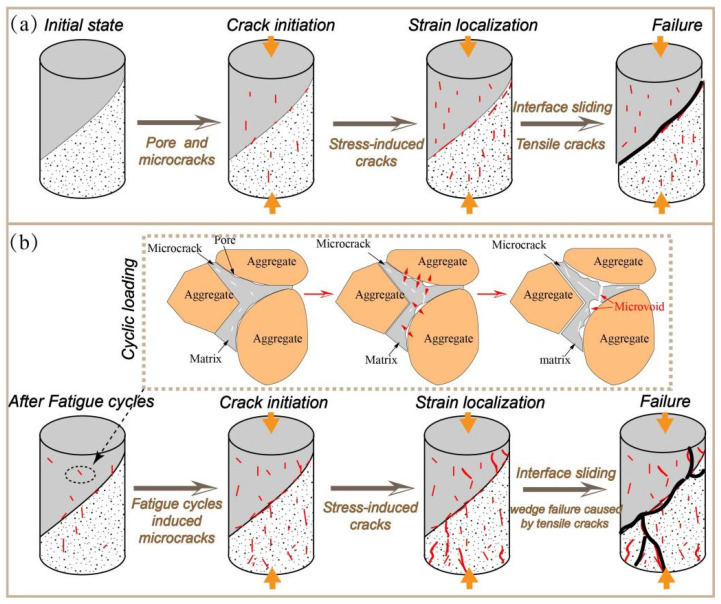
Crack propagation and analysis of the underlying mechanisms. (**a**) Before quasi-static cyclic loading. (**b**) After quasi-static cyclic loading.

To further elucidate the damage evolution mechanisms, this study takes the A45 composite as the key research object, as it represents an ideal transitional state featuring both tensile and shear failure. Integrating macroscopic observations and mesoscopic analysis (Figure 11), it systematically explains the failure process and damage accumulation behavior under cyclic loading. Under uniaxial static compression (Figure 11a), failure of the A45 specimen is highly localized along the predefined 45° weak interface, exhibiting typical brittle shear slip with concentrated energy release. The underlying mechanism is that during monotonic loading, once the shear stress generated by axial loading exceeds the interface shear strength, interfacial bonding fails instantaneously. This is reflected in the stress–strain curve as an abrupt post-peak stress drop, indicating a clear, single failure path. After quasi-static cyclic loading (Figure 11b), however, the failure mechanism shifts markedly toward a composite “interface slip–end tension” mode. The cyclic loading–unloading process does not cause immediate failure; instead, it gradually accumulates irreversible micro-plastic deformation and micro-crack propagation, continuously weakening the stiffness of the interfacial zone. When the specimen is subsequently subjected to monotonic loading, the original load-transfer path is reconfigured. Stress is no longer distributed solely along the single interface: a part continues to act on the weakened interface, while another portion is redistributed and strongly concentrated at geometric discontinuities, such as the concrete–rock wedge corners, forming high-stress concentration zones and new crack-initiation sites.

From a critical perspective, the limitations of the adopted methodologies must be acknowledged when interpreting these damage mechanisms. First, although the 3D-DIC technique provides high-precision full-field surface strain monitoring, it is fundamentally restricted to external surface observations and cannot directly capture the complex 3D internal crack propagation within the opaque rock–concrete composites. Second, the PFC2D numerical model represents a two-dimensional simplification of a naturally three-dimensional physical process. Consequently, out-of-plane stress redistributions and complex 3D spatial crack coalescences are inherently simplified. Future research integrating X-ray computed tomography (CT) and 3D discrete element modeling will be necessary to fully elucidate the spatial evolution of fatigue damage.

As damage progressively accumulates to a critical state, two failure mechanisms are simultaneously activated during subsequent loading: ongoing shear slip along the weakened interface, and tensile cracking induced in the stress-concentration zones. These two mechanisms interconnect and propagate in a coupled manner, eventually forming a composite wedge-shaped failure body. This progressive, stage-wise damage evolution is further corroborated by the cooperative propagation of shear and tensile cracks observed in PFC numerical simulations, as well as by the step-like increase in acoustic emission ring-down counts, confirming the dominant role of the composite damage mechanism during the fatigue process.

## 4. Analysis of Energy Evolution Laws

### 4.1. Variation in Cumulative Plastic Strain with Cycle Number

**Figure 12:**
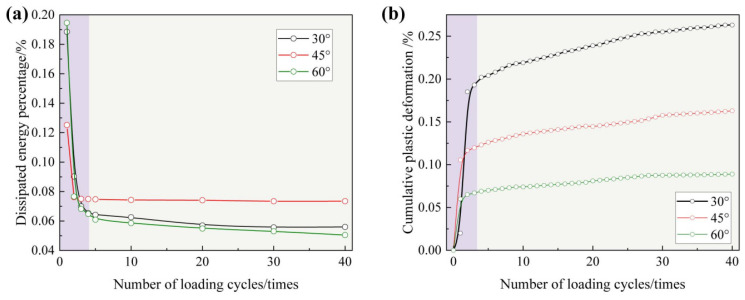
Plastic deformation of the composite after cyclic disturbance. (**a**) Dissipated energy ratio per cycle. (**b**) Cumulative plastic deformation.

The deformation and failure of rock–concrete composites under cyclic loading–unloading constitute an energy-driven instability process. Examining the evolution of energy characteristics after cyclic disturbance can therefore effectively reveal the underlying deformation mechanisms and damage behavior of the composites. Based on the calculations in Appendix A.3., Figure 12 respectively presents the evolution of dissipated energy and cumulative plastic strain from the 1st to the 40th cycle. The cumulative plastic strain for all three interface angles follows a characteristic trend of “rapid initial increase followed by progressive deceleration” (Figure 12a). Plastic strain accumulated relatively quickly over the first 1–2 cycles and then gradually slowed. After 40 cycles, the cumulative plastic strains reached 0.263% (30°), 0.1628% (45°), and 0.089% (60°). Notably, the plastic strain in the 30° specimen was approximately three times greater than that in the 60° specimen. Figure 12b further examines the variation in the dissipated-energy ratio with cycle number. The results show that the ratio decreases rapidly during the initial cycles before gradually stabilizing. The relatively high ratio in the first cycle is attributed primarily to energy consumption during fissure compaction, as aggregates in the upper concrete layer are compressed. With increasing cycles, the fissures progressively close and stabilize, leading to a decline in the dissipated-energy ratio until it plateaus at a low level.

### 4.2. Analysis of Energy Conversion and Distribution in the Composite

**Figure 13:**
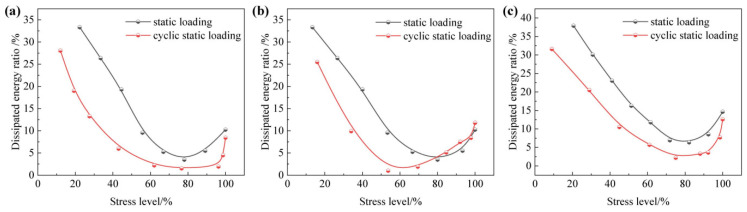
Analysis of energy density curves of rock–concrete composite before and after fatigue disturbance: (**a**) 30°, (**b**) 45°, (**c**) 60°.

The energy response of specimens under fatigue cycling at various stress levels provides an effective indicator of internal damage evolution during loading [35]. As illustrated in Figure 13, the evolution trends of energy characteristics are generally similar across different interface angles, with the elastic strain energy density being nearly equivalent to the total energy density. Specimens that underwent quasi-static cyclic loading demonstrated higher total and elastic strain energy densities than their non-fatigued counterparts. Specifically, the total energy densities for A30, A45, and A60 increased from 48.6 MJ·mm^−3^, 33.2 MJ·mm^−3^, and 12.0 MJ·mm^−3^ to 60.7 MJ·mm^−3^, 54.2 MJ·mm^−3^, and 23.7 MJ·mm^−3^, respectively, after cyclic disturbance. This anomalous enhancement is attributed to the compaction effect and cyclic hardening of geomaterials. The relatively low cyclic stress closes initial micro-defects within the matrices and interfaces rather than inducing macroscopic failure. This microstructural densification temporarily enhances the overall stiffness, enabling the composite to withstand higher peak stresses and store more elastic strain energy during subsequent monotonic loading. Additionally, the energy storage capacity decreases significantly with an increasing interface angle, highlighting the crucial role of interface geometry in energy distribution.

**Figure 14:**
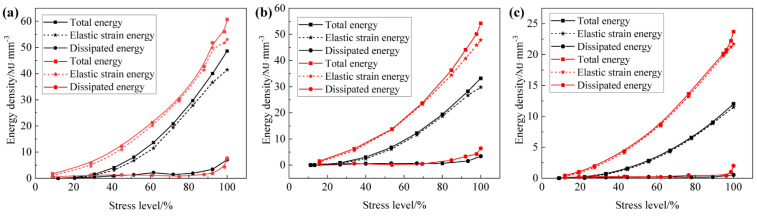
Energy proportion analysis of rock–concrete composite before and after fatigue disturbance: (**a**) 30°, (**b**) 45°, (**c**) 60°.

Analyzing the dissipated energy ratio elucidates the material’s energy absorption and release behavior under cyclic stress, thereby enabling an assessment of its fatigue resistance and dissipation efficiency, as well as predictions of service life and compressive strength evolution. Figure 14 shows that at low stress levels, specimens with prior cyclic loading history possess a lower dissipated energy ratio than those under static conditions, reflecting a higher elastic storage capacity and reduced energy loss. This suggests that low-stress cyclic loading promotes microstructural stabilization, which in turn enhances energy storage. In contrast, as the stress level rises, the dissipated energy ratio for the cyclically loaded 45° composite eventually surpasses that under static loading, marking the onset of damage accumulation effects. This accumulated damage stems primarily from the initiation and growth of internal microcracks, coupled with increased plastic deformation.

### 4.3. Energy Conversion Threshold and Discussion

**Figure 15:**
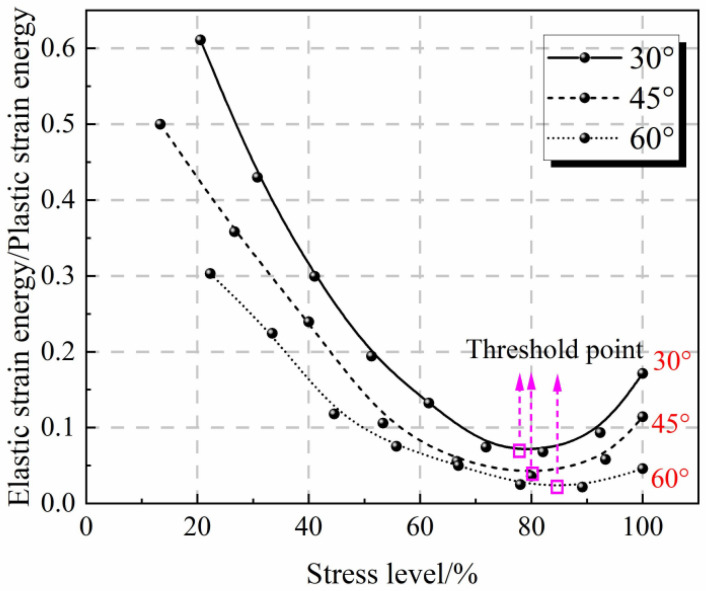
Changes in energy dissipation coefficient of specimens after fatigue static loading.

The evolution of the energy consumption coefficient—defined as the ratio of elastic strain energy to plastic strain energy—provides a clear indication of internal damage progression in rock–concrete composites [36,37]. It is calculated by integrating the area under the stress–strain curve up to the point of macroscopic failure. This dimensionless threshold represents the ultimate energy storage limit of the composite before the internal energy dissipation mechanism abruptly shifts to drive unstable crack propagation. As shown in Figure 15, during early loading the coefficient first decreases and then rises. At low stress levels, the material displays strong elastic recovery, storing most of the input energy elastically with little plastic deformation or dissipation. As stress nears the critical level, however, frictional slip along fissures and accumulated damage intensify, causing energy dissipation to rise markedly and elastic storage capacity to decline. Consequently, the specimen enters the plastic deformation stage and approaches failure.

The energy distribution thresholds for composites with different interface angles are approximately 78% (A30), 80% (A45), and 83% (A60). This progressive increase is linked to fundamental changes in the interfacial stress state. Under axial compression, higher interface angles produce a more pronounced rise in the shear stress component resolved along the interface relative to the normal component. The resulting shear stress enhances frictional resistance and interlocking before large-scale slip occurs, allowing a larger fraction of the input energy to be stored elastically. Hence, a higher stress (or energy density) is needed to overcome this resistance and initiate substantial plastic deformation and damage, leading to the observed rise in the energy threshold with increasing interface angle. From a practical engineering perspective, these energy conversion thresholds hold significant application value. In the stability assessment of open-pit slope support systems, this threshold can serve as a quantitative safety criterion and an early-warning indicator. When real-time field monitoring data (such as micro-seismic energy release or local strain accumulation) reveals that the internal energy state of the rock–concrete structure is approaching its specific threshold (e.g., 78% for a 30° interface or 83% for a 60° interface), it alerts engineers that the structure is on the verge of transitioning from stable energy storage to unstable macroscopic failure. This provides a crucial time window to implement timely reinforcement measures or evacuate personnel, thereby preventing catastrophic slope failures.

Despite macroscopic stiffness degradation caused by fatigue-induced microcracking, the composite exhibits enhanced elastic energy storage due to micro-structural stress redistribution. Pre-fatigue loading preferentially damages weak links, such as interfacial zones. During subsequent compression, these microcracks undergo secondary closure, increasing internal friction. Concurrently, the applied stress transfers to the higher-strength, intact matrix. Together, this enhanced crack-face friction and stress redistribution raise the energy barrier for unstable crack propagation. Consequently, the specimen must store greater elastic strain energy to overcome this internal resistance before ultimate failure.

## 5. Conclusions

This study systematically investigated the influence of interface angle on the mechanical and fracture characteristics of rock–concrete composites subjected to fatigue damage. The main conclusions are summarized as follows:

(1) The peak compressive strength of the rock–concrete composite degrades markedly as the interface angle increases. Both the strength loss rate and the brittleness index increase significantly after cyclic disturbance. Specimens with larger interface angles exhibit higher equivalent elastic moduli and substantially reduced cumulative plastic deformation, indicating a macroscopic shift in the deformation mechanism from “plastic–dissipation–progressive–instability” to “elastic–storage–sudden–instability”.

(2) Under monotonic static loading, the dominant failure mode shifts from through–thickness tensile failure at 30° to interfacial slip failure at 45° and 60°. The application of cyclic disturbance further modifies the damage evolution: the 30° specimens develop a dense internal network of tensile cracks, whereas the 45° and 60° specimens evolve into a combined failure mode characterized by interfacial shear slip accompanied by tensile cracking at the specimen ends.

(3) Cyclic disturbance increases both the total energy density and the elastic strain energy density of the composite while reducing the dissipated-energy ratio. The cumulative plastic strain exhibits rapid initial growth followed by progressive deceleration as the number of cycles increases. After cyclic disturbance, the material’s energy storage capacity is enhanced, and the dissipated-energy fraction is lowered. The energy-conversion threshold explicitly increases with the interface angle, indicating that higher inclination angles effectively extend the elastic deformation stage.

(4) Increasing the interface angle enhances geometric interlocking and reorganizes the internal force-chain network from an approximately isotropic distribution to a highly aligned configuration. This meso-scale reorganization of load-transfer pathways produces pronounced stress concentrations at the interface and drives a macroscopic transition of the failure mode from tensile-dominated to shear-dominated, manifested by a progressive increase in the proportion of shear cracks.

While this study clarifies the fatigue damage mechanisms of rock–concrete composites, certain limitations exist. The laboratory-scale setup, limited pre-fatigue cycles, and 2D numerical simplifications restrict the comprehensive evaluation of field-scale effects, high-cycle fatigue, and 3D crack propagation. Future work will address these gaps through large-scale experiments, high-cycle fatigue testing, and 3D discrete element modeling coupled with X-ray CT scanning.

## Figures and Tables

**Table 1:** Specimen numbers and test settings.

Group	Interface Angle	Number of Cycles/Times	50% σ_max_/kN
A30-0	30°	0	-
A30-40		40	300
A45-0	45°	0	-
A45-40		40	240
A60-0	60°	0	-
A60-40		40	120

Note: The target stress upper limits for the cyclic loading phase (i.e., 300, 240, and 120 kN, corresponding to 50% σ_max_) were strictly derived from preliminary monotonic static loading tests.

**Table 2:** Mesoscopic parameters for the rock and concrete materials in the discrete element model.

Material	Density (kg/m^3^)	Elastic Modulus (GPa)	Poisson’s Ratio	UCS (MPa)	Tensile Strength (MPa)	Stiffness Ratio	Normal Bond Strength (MPa)	Shear Bond Strength (MPa)	Friction Angle (°)
Rock	2650.00	15.00	0.20	75.00	7.50	3.60	30.20	19.10	37.70
Concrete	3100.00	25.00	0.22	45.00	4.50	1.30	1.20	1.10	35.00

**Figure A1:**
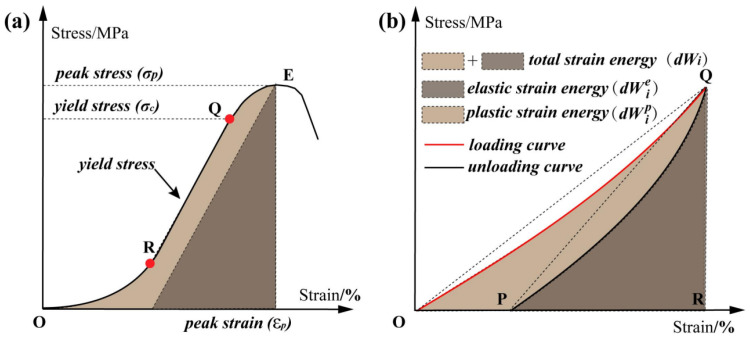
Schematic diagram of mechanical parameter calculation: (**a**) schematic diagram for brittleness index calculation; (**b**) schematic diagram of the stress–strain curve and energy density calculation for the i-th cycle.

## Data Availability

The original contributions presented in this study are included in the article. Further inquiries can be directed to the corresponding authors.

## Appendix A

### Appendix A.1. Calculation of Brittleness Index

The brittleness index is determined by comparing the cumulative elastic strain energy obtained from the pre-peak segment of the stress–strain curve with the cumulative strain energy corresponding to an ideal linear-elastic deformation (Figure A1a). It should be clarified that rock–concrete composites are inherently quasi-brittle, manifesting pre-peak nonlinearity and plasticity. Strictly speaking, the releasable elastic strain energy should be calculated using the actual unloading modulus. However, since determining the precise unloading modulus at peak stress is experimentally challenging, approximating it with the initial elastic modulus (E) is a widely accepted and standardized theoretical assumption in classical rock energy dissipation theories. This formula effectively isolates the idealized releasable elastic component from the total input energy for comparative analysis. The calculation is expressed as follows [35,36]:

(A1) $$BI=\frac{dW_{\mathrm{et}}}{dW_{\mathrm{et}}+dW_{p}}$$

(A2) $$dW_{p}=dW_{t}-dW_{et}=\int_{0}^{p}\sigma d\varepsilon -\frac{1}{2E}\sigma_{p}^{2}$$

(A3) $$dW_{et}=\frac{1}{2E}\sigma_{p}^{2}$$

In the formulae, *dW_i_* represents the total deformation energy; *dW_ie_* denotes the elastic deformation energy of the load; *dW_ip_* indicates the plastic deformation energy of the load.

### Appendix A.2. Stress–Strain Curve for the i-th Cycle and Energy Density Calculation

Figure A1b shows the stress–strain response during the i-th cycle, where segment *O–Q* denotes the loading phase and segment *Q–P* denotes the unloading phase. For a single loading–unloading cycle, the deformation moduli of the composite material are calculated as follows [37]:

(A4) $$E_{\mathrm{loading}}=\frac{\sigma }{\varepsilon_{R}}=\frac{QR}{OR}$$

(A5) $$E_{\mathrm{unloading}}=\frac{\sigma }{\varepsilon_{R}-\varepsilon_{P}}=\frac{QR}{PR}$$

In the above equations, *E_loading_* denotes the loading deformation modulus and *E_unloading_* the unloading deformation modulus. Point *Q* corresponds to the peak-stress point of each cycle; *ε_p_* denotes the residual (permanent) strain per cycle, and (*ε_R_* − *ε_P_*) represents the elastic strain recovered during unloading in each cycle.

### Appendix A.3. Calculation of Energy Parameters for the i-th Cycle

The energy parameters for the *i*-th cycle are calculated as follows [37]:

(A6) $$dW_{i}=\int_{0}^{\varepsilon_{R}}\sigma d\varepsilon$$

(A7) $$dW_{i}^{e}=\int_{\varepsilon_{P}}^{\varepsilon_{R}}\sigma d\varepsilon$$

(A8) $$dW_{i}^{p}=dW_{i}-dW_{i}^{e}=\int_{0}^{\varepsilon_{R}}\sigma d\varepsilon -\int_{\varepsilon_{P}}^{\varepsilon_{R}}\sigma d\varepsilon$$

In the formulae, *ε_R_* is the total strain; *ε_P_* is the plastic strain; (*ε_R_* − *ε_P_*) represents the elastic strain.
